# The Volatile Signature: Tracking Ripening Dynamics to Ensure Goat Cheese Quality

**DOI:** 10.3390/s26051583

**Published:** 2026-03-03

**Authors:** Giovanni Ferrara, Cristina Matarazzo, Maria Staiano, Sabato D’Auria, Rosaria Cozzolino

**Affiliations:** 1 Istituto di Scienze dell’Alimentazione, Consiglio Nazionale delle Ricerche, 83100 Avellino, Italy; giovanniferrara1@cnr.it (G.F.); maria.staiano@cnr.it (M.S.); rosaria.cozzolino@isa.cnr.it (R.C.); 2 Department of Agriculture, Environmental and Food Sciences, University of Molise, 86100 Campobasso, Italy; c.matarazzo3@studenti.unimol.it

**Keywords:** goat cheese, ripening, volatile organic compounds, HS-SPME/GC–MS, aroma profile, sensors

## Abstract

Cheese ripening involves a series of biochemical and microbiological transformations that directly affect the texture, aroma, flavor, and quality of the final product. This study aimed to characterize the volatile organic compounds (VOCs) produced during the ripening of goat cheese to find suitable molecular markers for monitoring the maturation process. Headspace solid-phase microextraction coupled with gas chromatography-mass spectrometry (HS-SPME/GC–MS) was applied to samples collected at different ripening times (0, 30, 60, 90, 120, and 150 days). Overall, sixty-eight different VOCs were identified, including alcohols, esters, ketones, carboxylic acids, aldehydes, terpenes, sulfur compounds, and others. The total volatile content progressively increased up to 120 days and slightly decreased thereafter. This dynamic evolution reflected the interplay of proteolysis, lipolysis, and microbial metabolism occurring during the ripening process. Among the compounds, 2-butanone and 2-butanol appeared as promising volatile markers of the advanced ripening stages. These results offer new insights into goat cheese flavor development and support the design of a sensing approach for a first warning of the end of the cheese maturation process.

## 1. Introduction

Cheese is a dairy matrix formed through the curdling of milk, cream, or skimmed buttermilk obtained from various animal species, such as bovine and caprine. Cheese can be classified as either fresh or fermented depending on its processing, which involves a complex interaction of microbiological and biochemical transformations that, beginning at the first stages of cheesemaking, continue across the maturation [1].

Cheesemaking typically begins with the addition of starter lactic acid bacteria (SLAB), which activate the fermentation by breaking down milk carbohydrates (in particular, lactose), proteins, and fats into simpler compounds [2]. SLAB are sometimes intentionally added to acidify the milk and facilitate curd formation. Once the curd is produced, the whey is drained, and the curd is prepared for maturation, which represents the crucial step in defining the final cheese characteristics [1,3,4].

During ripening, cheese develops its unique biochemical and nutritional attributes, but also its peculiar texture, aroma, and flavor profile. Maturation starts at once after curd formation and can last from several days to many months, depending on the type of cheese [3,4]. During this phase, conditions, including temperature and humidity, can be carefully controlled to tailor the desired quality attributes of the final product [5]. In addition to SLAB, non-starter lactic acid bacteria (NSLAB) also play a key role in cheese ripening. Compared to SLAB, which dominate the early fermentation stage, NSLAB, often naturally present in raw milk or introduced via the environment, increase during cheese aging. The presence of NSLAB is particularly significant in long-ripened cheeses and affects the development of complex cheese flavors and textures [2,6]. During the process of ripening, a range of physicochemical and enzymatic reactions occur, which must be carefully controlled to produce cheese that is safe, stable, and appealing in taste, texture, and aroma. Specifically, enzymes from milk, SLAB, and NSLAB break down macronutrients into smaller compounds, including peptides, free amino acids, fatty acids, and other secondary metabolites [7,8,9].

Among them, VOCs are the primary contributors to cheese aroma and flavor. These compounds are produced through different biochemical pathways, including lipolysis, proteolysis, and fermentation of citrate and residual sugars. Their qualitative and quantitative profile reflects the combination of the enzymatic and microbial activity occurring during cheese maturation, directly influencing the bouquet of the final product. Given their dynamic nature, VOCs have been successfully utilized as putative molecular markers of cheese ripening, offering valuable information on the stage of maturation and on the potential sensory defects or quality deviations of cheese [10,11,12,13].

In flavor research, HS-SPME/GC is the most used analytical methodology to separate and identify volatile compounds. In fact, HS-SPME is a relatively simple, solventless, high-sensitivity, and high-throughput automated sampling method that is currently considered the most suitable approach to extract the volatile contributors to aroma in numerous food matrices [14,15]. This technique is also widely utilized to profile VOCs in dairy products due to its high sensitivity for the identification of low concentrations of odorant compounds such as sulfur compounds in different varieties of cheese [16,17,18].

To characterize the evolution of aroma compound patterns and identify VOCs as putative markers of cheese ripening, we investigated the VOC profile by headspace solid-phase microextraction (HS-SPME) coupled with gas chromatography-mass spectrometry (GC–MS) during the ripening process of a goat cheese prepared ad hoc for this study. The identified compounds have been used to design a preliminary colorimetric sensor for a first warning of the end of the cheese maturation process. This approach aligns with the growing field of chemosensors designed for quality control, where VOCs serve as specific metabolic markers [12,19].

In the dairy industry, the development of gas-phase sensors enables the non-destructive monitoring of cheese ripening by detecting key maturation indicators [20,21]. Furthermore, the integration of smartphone-based qualitative colorimetric assays could offer a portable, naked-eye alternative for an early warning facilitating the evaluation of food aging processes.

## 2. Materials and Methods

### 2.1. Cheese Samples

Goat cheese was produced from a single batch of milk in a local dairy, yielding six samples of 500 g each. The cheese was ripened by the local dairy under controlled conditions for the entire duration of the experiments and until analyses, which were performed at 0, 30, 60, 90, 120, and 150 days.

### 2.2. Sample Preparation and HS-SPME Procedure

Cheese samples (7 g for each sample) were finely grated into small particles and immediately stored at −80 °C in 20 mL headspace vials (Supelco, Bellefonte, PA, USA) fitted with a Teflon (PTFE) septum and an aluminum cap (Chromacol, Hertfordshire, UK) until the analyses were performed. VOC analysis was conducted according to Cozzolino et al. [18]. For sample preparation, thawed cheese samples were spiked with 10 μL 3-octanol (0.4 μg/μL) (Sigma-Aldrich Co., St. Louis, MO, USA), used as internal standard (IS) to ensure analytical reproducibility. For the release of VOCs, the vial was held at 40 °C for 30 min (equilibration step). Subsequently, a divinylbenzene/carboxen/poly-dimethylsiloxane (DVB/CAR/PDMS) fiber was automatically inserted into the vial for 60 min to allow adsorbing of VOCs onto the SPME fiber surface. Before the first daily analysis, the fiber was conditioned for 5.0 min at the operating temperature of the GC injector port.

### 2.3. GC–MS Analysis

The SPME fiber was automatically inserted into the injector port of the gas chromatograph device, model GC 7890A (Agilent Technologies, Santa Clara, CA, USA), equipped with a quadrupole mass spectrometer 5975 C (Agilent Technologies), where VOCs were thermally desorbed for 10 min at 240 °C and directly transported to a capillary column HP-Innowax (30 m × 0.25 mm × 0.5 µm Agilent J&W) for the analysis. The oven temperature was initially set at 40 °C for 2 min, ramped at 5 °C/min to 65 °C and kept at 65 °C for 2 min, then increased at 10 °C/min to 240 °C and held for 9 min at 240 °C. The temperatures of the ion source and the quadrupole were 230 °C and 150 °C, respectively, and helium, at a flow rate of 1.5 mL/min, was used as the carrier gas. The pulsed splitless mode was set for the analysis and mass spectra were taken at an ionization energy of 70 eV. VOCs were identified by mass selective detector, operating in the mass range 30–300 u with a scan rate of 2.7 scans/s. Each replicate was analyzed in triplicate in a randomized sequence where blanks were also run. VOCs were identified by comparing the mass spectra in the available databases (NIST-2014/Wiley 7.0 libraries) by matching the calculated LRI with literature data and by evaluating their retention times with those of commercial standards, when available. The areas of the identified volatiles were extrapolated from the total ion current (TIC) and semi-quantitative data of each metabolite (Relative Peak Area, RPA%) were obtained by comparison with the peak area of the IS.

### 2.4. Materials and Reagents

All reaction mixtures were prepared according to the method described by Prakobdi and Saetear [22]. Briefly, 12% (*w*/*v*) of sodium hydroxide was prepared by dissolving 12.0 g of NaOH (Sigma-Aldrich Co., St. Louis, MO, USA) in 100 mL of deionized water. A 3% (*w*/*v*) iodine solution was prepared by dissolving 3.0 g of iodine pellets (I_2_, Sigma-Aldrich Co., St. Louis, MO, USA) and excess of potassium iodide (KI, Molekula Ltd., Darlington, UK) into 100 mL of deionized water. 2-Butanone and ethanol were purchased from Sigma-Aldrich (Sigma-Aldrich Co., St. Louis, MO, USA) and AppliChem (Darmstadt, Germany), respectively.

Each experimental setup consisted of two open 2 mL glass vials placed inside a 100 mL glass bottle. One vial contained 0.33 mL of 12% (*w*/*v*) NaOH and the second vial contained 0.33 mL of an aqueous 2-butanone solution (200 mM). The concentration of 2-butanone (200 mM) was selected for the iodoform test, as it mimics the amount of this compound in 1 Kg of goat cheese, according to the data reported in Table 1 (see Section 3.1).

The NaOH-containing vial was exposed to volatile compounds for 30, 60, 120, and 180 min at room temperature. After exposure, 0.67 mL of 3% iodine solution was added to the NaOH vial to initiate the iodoform reaction. Control experiments were performed by exposing NaOH to water vapor and ethanol vapor (200 mM) under identical conditions.

### 2.5. Spectrophotometric Characterization

To provide analytical evidence of the precipitation kinetics, turbidity measurements were performed using a Jasco V-750 spectrophotometer (Jasco Corporation, Tokyo, Japan). The reaction mixtures (0.33 mL NaOH 12% *w*/*v* exposed at different times to 2-butanone vapors) added with 0.67 mL I_2_ (3% *w*/*v*) were analyzed at fixed wavelength mode at 1000 nm using a 1.0 cm quartz cuvette [22]. As baseline, a freshly prepared and non-exposed mixture was used.

### 2.6. Densitometric Analysis

To monitor the iodoform precipitation by analyzing the color intensity, a digital densitometry measurement was performed using the ImageJ software (version 1.51), a well-known and established open-source software for image processing [23,24]. A smartphone was used to capture image of the observed color changes within the reaction vials. Following the methodology described by Kubheka et al. [25], the Mean Gray Value (MGV), which represents the mean grayscale intensity, was measured by defining a rectangular Region of Interest (ROI) within the central portion of the vials to capture the yellow precipitate. The software calculates the MGV, providing the quantification of the reaction progress based on the relationship between color information and precipitate density. The NaOH solution (12% *w*/*v*) with the addition of I_2_ solution (3% *w*/*v*) was used as the reference blank to provide the baseline. To quantify the progression of the reaction during the exposition time, the absorbance values were derived using a baseline subtraction method (MGV_BLANK_-MGV_SAMPLE_) to ensure that the decrease in pixel brightness caused by the accumulation of the yellow precipitate is converted into a positive correlation of the reaction progress.

### 2.7. Statistical Analysis

A Principal Component Analysis (PCA) was performed on overall HS-SPME/GC-MS data obtained from the six goat cheese samples at different ripening stages, to evaluate the similarities or dissimilarities of the VOC profile of the goat cheeses along with aging. Data were expressed as the mean ± standard deviation and statistical analyses were performed using the SPSS software package, Version 19.0 (SPSS Inc., Chicago, IL, USA), and Origin 2018 software (OriginLab, Northampton, MA, USA).

## 3. Results and Discussion

### 3.1. VOC Analysis

HS-SPME/GC-MS analysis of goat cheese samples ripened at different times allowed the identification of a total of 68 VOCs, belonging to eight different classes, such as alcohols (17), esters (17), carboxylic acids (11), ketones (9), aldehydes (6), terpenes (4), sulfurs (3), and other (1), as reported in Table 1.

**Table 1:** VOC concentration (mg/kg) in goat cheese during ripening expressed as mean ± standard deviation. ND indicates compounds not detected.

		Ripening Time					
Compound	Code	Day 1	Day 30	Day 60	Day 90	Day 120	Day 150
**Alcohols**							
Ethanol	Alc1	0.21 ± 0.01	2.70 ± 0.03	6.26 ± 0.01	12.35 ± 0.92	19.80 ± 0.09	3.71 ± 0.08
2-butanol	Alc2	ND	ND	ND	0.10 ± 0.03	0.14 ± 0.01	6.36 ± 0.36
1-Propanol-2-methyl (Isobutyl alcohol)	Alc3	ND	0.11 ± 0.00	0.09 ± 0.00	0.16 ± 0.01	ND	0.04 ± 0.01
2-Pentanol	Alc4	ND	ND	ND	ND	ND	0.10 ± 0.01
1-Butanol	Alc5	ND	0.01 ± 0.00	0.04 ± 0.00	0.08 ± 0.01	0.15 ± 0.00	0.08 ± 0.01
1-Butanol-3-methyl (Isoamyl alcohol)	Alc6	0.40 ± 0.01	1.36 ± 0.01	1.38 ± 0.00	2.08 ± 0.10	2.17 ± 0.03	0.64 ± 0.11
1-Pentanol	Alc7	0.01 ± 0.00	0.01 ± 0.00	0.03 ± 0.00	0.01 ± 0.01	ND	ND
2-Heptanol	Alc8	ND	0.06 ± 0.00	0.05 ± 0.00	0.10 ± 0.00	0.15 ± 0.01	0.23 ± 0.00
1-Hexanol	Alc9	0.01 ± 0.00	ND	0.04 ± 0.00	0.10 ± 0.00	0.11 ± 0.00	0.06 ± 0.01
2-Ethyl-1-hexanol	Alc10	0.03 ± 0.00	0.06 ± 0.00	0.12 ± 0.00	0.14 ± 0.01	0.24 ± 0.01	0.07 ± 0.00
2-Nonanol	Alc11	ND	0.03 ± 0.00	0.06 ± 0.00	0.10 ± 0.00	0.18 ± 0.01	0.13 ± 0.04
1-Octanol	Alc12	0.01 ± 0.00	ND	0.05 ± 0.00	ND	ND	0.09 ± 0.02
2,3-Butanediol	Alc13	ND	ND	0.14 ± 0.00	0.65 ± 0.00	1.72 ± 0.01	0.53 ± 0.01
2-Furanmethanol	Alc14	0.01 ± 0.00	0.01 ± 0.00	ND	0.02 ± 0.02	0.11 ± 0.00	0.03 ± 0.01
1-Propanol, 3-(methylthio)-	Alc15	ND	0.02 ± 0.00	0.03 ± 0.00	0.05 ± 0.00	ND	0.04 ± 0.01
Benzyl alcohol	Alc16	ND	0.01 ± 0.00	0.03 ± 0.00	0.07 ± 0.00	0.08 ± 0.00	0.03 ± 0.01
Phenethyl alcohol	Alc17	0.03 ± 0.00	0.27 ± 0.00	0.46 ± 0.00	0.65 ± 0.04	0.89 ± 0.07	0.24 ± 0.04
**Ketones**							
2-Propanone	K1	0.06 ± 0.01	0.05 ± 0.00	0.37 ±0.00	0.11 ± 0.01	0.22 ± 0.02	0.03 ± 0.00
2-butanone	K2	0.02 ± 0.00	0.03 ± 0.00	0.14 ± 0.00	0.39 ± 0.05	0.32 ± 0.02	6.35 ± 0.07
2-Pentanone	K3	ND	0.06 ± 0.00	0.26 ± 0.00	0.29 ± 0.02	0.38 ± 0.03	0.20 ± 0.04
2,3-Butanedione (diacetyl)	K4	0.08 ± 0.00	0.12 ± 0.01	0.29 ± 0.00	0.36 ± 0.02	0.32 ± 0.02	0.07 ± 0.01
2-Heptanone	K5	0.02 ± 0.00	0.09 ± 0.00	1.39 ± 0.01	0.64 ± 0.27	0.99 ± 0.02	0.67 ± 0.03
2-Nonanone	K6	0.01 ± 0.00	0.09 ± 0.02	0.55 ± 0.01	0.57 ± 0.05	1.05 ± 0.03	0.94 ± 0.02
3-Hydroxy-2-butanone (Acetoin)	K7	0.65 ± 0.04	1.41 ± 0.08	1.34 ± 0.00	1.82 ± 0.02	1.44 ± 0.04	0.48 ± 0.00
2-Octanone	K8	ND	ND	0.03 ± 0.00	0.02 ± 0.00	ND	0.02 ± 0.00
8-Nonen-2-one	K9	ND	ND	0.06 ± 0.00	0.05 ± 0.00	ND	0.05 ± 0.01
**Aldehydes**							
2-Methylpropanal	Ald1	0.02 ± 0.00	0.02 ± 0.00	ND	ND	ND	ND
2-Methylbutanal	Ald2	0.02 ± 0.00	0.01 ± 0.00	ND	0.05 ± 0.00	0.07 ± 0.01	ND
3-Methylbutanal	Ald3	0.25 ± 0.02	0.13 ± 0.00	0.16 ± 0.00	0.22 ± 0.00	0.29 ± 0.04	0.19 ± 0.01
Nonanal	Ald4	0.01 ± 0.00	ND	0.06 ± 0.00	0.12 ± 0.01	0.27 ± 0.00	0.05 ± 0.01
Benzaldehyde	Ald5	0.01 ± 0.00	0.01 ± 0.00	0.04 ± 0.00	0.07 ± 0.01	0.11 ± 0.01	0.04 ± 0.00
Phenylacetaldehyde (Benzenacetaldehyde)	Ald6	0.03 ± 0.00	0.07 ± 0.00	0.07 ± 0.00	0.13 ± 0.04	0.29 ± 0.00	0.09 ± 0.01
**Esters**							
Ethyl acetate	E1	0.04 ± 0.00	0.16 ± 0.00	0.11 ± 0.00	0.20 ± 0.00	0.48 ± 0.01	0.11 ± 0.01
Ethyl butyrate	E2	0.01 ± 0.00	0.24 ± 0.00	0.26 ± 0.00	0.80 ± 0.00	1.14 ± 0.07	0.64 ± 0.02
Ethyl 3-methylbutyrate	E3	0.01 ± 0.00	0.02 ± 0.00	0.05 ± 0.00	0.08 ± 0.01	0.16 ± 0.01	0.03 ± 0.01
Isoamyl acetate	E4	ND	0.02 ± 0.00	0.05 ± 0.00	0.10 ± 0.01	0.21 ± 0.00	0.06 ± 0.03
Ethyl pentanoate	E5	ND	0.04 ± 0.00	0.02 ± 0.00	0.03 ± 0.00	ND	0.02 ± 0.00
Isobutyl butyrate	E6	ND	0.01 ± 0.00	0.02 ± 0.00	0.02 ± 0.00	ND	ND
Ethyl 2-butenoate	E7	ND	0.01 ± 0.00	0.02 ± 0.00	0.02 ± 0.00	ND	ND
Pentyl acetate	E8	ND	0.01 ± 0.00	ND	ND	ND	ND
Isobutyl isopentanoate	E9	ND	0.04 ± 0.00	ND	0.08 ± 0.01	ND	ND
Ethyl hexanoate	E10	0.01 ± 0.00	3.85 ± 0.04	1.30 ± 0.00	2.75 ± 0.05	4.42 ± 0.05	2.04 ± 0.03
Ethyl 3-hexenoate	E11	ND	0.20 ± 0.01	0.03 ± 0.00	0.02 ± 0.00	ND	ND
Isoamyl butyrate	E12	ND	0.04 ± 0.00	0.06 ± 0.00	0.08 ± 0.01	0.22 ± 0.01	0.08 ± 0.00
Ethyl heptanoate	E13	ND	0.01 ± 0.00	0.02 ± 0.00	0.03 ± 0.00	0.06 ± 0.01	0.03 ± 0.00
Isobutyl hexanoate	E14	ND	0.11 ± 0.01	ND	0.03 ± 0.03	ND	0.02 ± 0.00
Ethyl octanoate	E15	ND	0.15 ± 0.02	0.20 ± 0.00	1.34 ± 0.02	2.18 ± 0.03	1.18 ± 0.02
Isoamyl hexanoate	E16	ND	0.06 ± 0.00	ND	0.02 ± 0.02	0.05 ± 0.00	ND
Ethyl decanoate	E17	ND	ND	0.03 ± 0.00	0.10 ± 0.01	0.21 ± 0.00	0.04 ± 0.01
**Carboxylic acids**							
Acetic acid	A1	0.12 ± 0.00	1.45 ± 0.06	1.35 ± 0.00	4.01 ± 0.06	8.44 ± 0.02	3.97 ± 0.07
Propanoic acid	A2	ND	0.03 ± 0.00	ND	0.15 ± 0.05	0.26 ± 0.00	0.09 ± 0.01
2-Methylpropanoic acid	A3	ND	0.23 ± 0.01	0.24 ± 0.00	0.42 ± 0.03	ND	0.42 ± 0.06
Butanoic acid	A4	0.03 ± 0.00	1.54 ± 0.06	2.87 ± 0.00	7.88 ± 0.85	11.11 ± 0.03	7.06 ± 0.05
3-Methyl-butyric acid	A5	0.01 ± 0.00	2.02 ± 0.13	3.03 ± 0.00	4.78 ± 0.46	6.42 ± 0.04	1.45 ± 0.01
Pentanoic acid	A6	ND	0.02 ± 0.00	0.03 ± 0.00	0.07 ± 0.00	0.10 ± 0.00	0.08 ± 0.01
Hexanoic acid	A7	0.17 ± 0.02	1.77 ± 0.04	5.75 ± 0.01	12.11 ± 0.05	20.10 ± 0.41	14.27 ± 0.44
Heptanoic acid	A8	ND	0.01 ± 0.00	0.05 ± 0.00	0.10 ± 0.00	0.20 ± 0.00	0.14 ± 0.01
Octanoic acid	A9	0.07 ± 0.00	0.24 ± 0.01	1.17 ± 0.00	2.88 ± 0.23	5.94 ± 0.02	4.30 ± 0.08
Nonanoic acid	A10	ND	ND	ND	ND	0.09 ± 0.00	0.03 ± 0.01
Decanoic acid	A11	0.01 ± 0.00	0.040 ±0.00	0.11 ± 0.00	0.20 ± 0.02	0.55 ± 0.00	0.21 ± 0.01
**Terpenes**							
α-Pinene	T1	0.03 ± 0.00	0.06 ± 0.00	0.16 ± 0.00	0.21 ± 0.01	0.24 ± 0.02	0.09 ± 0.01
β-Phellandrene/Sabinene	T2	0.01 ± 0.00	0.02 ± 0.00	0.05 ± 0.00	0.05 ± 0.00	ND	0.02 ± 0.01
Limonene	T3	0.03 ± 0.00	0.03 ± 0.00	0.09 ± 0.00	0.08 ± 0.01	0.53 ± 0.02	0.11 ± 0.01
p-Cymene	T4	0.01 ± 0.00	0.01 ± 0.00	0.03 ± 0.00	0.03 ± 0.00	ND	0.02 ± 0.00
**Sulfurs**							
Dimethyl disulfide	S1	0.02 ± 0.00	0.03 ± 0.00	ND	ND	ND	ND
Butanenitrile, 4-(methylthio)-	S2	0.02 ± 0.00	0.06 ± 0.00	0.11 ± 0.00	0.15 ± 0.01	0.23 ± 0.00	0.09 ± 0.00
Dimethyl sulfone	S3	0.01 ± 0.00	0.03 ± 0.00	0.04 ± 0.00	0.05 ± 0.00	0.15 ± 0.01	0.08 ± 0.02
**Others**							
Styrene	O1	ND	0.03 ± 0.00	0.10 ± 0.00	0.18 ± 0.01	0.24 ± 0.01	0.11 ± 0.00

**Figure 1:**
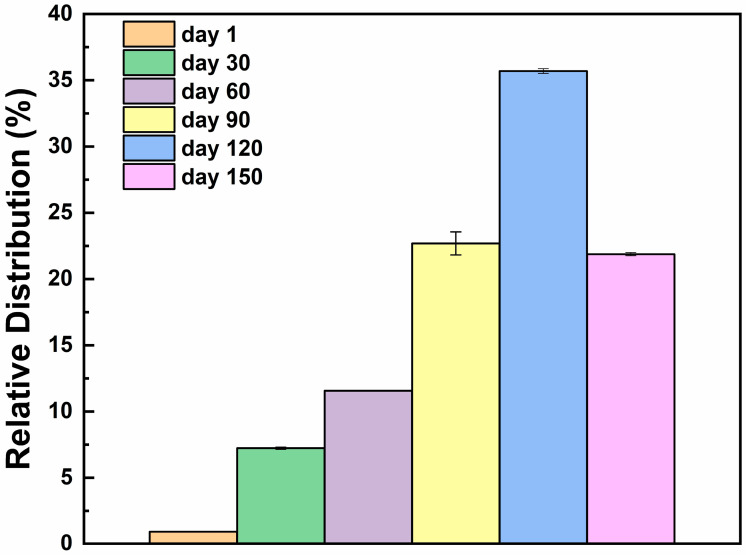
Relative distribution (%) of the total VOCs detected in the cheese samples at different ripening times: day 1 (orange), day 30 (green), day 60 (purple), day 90 (yellow), day 120 (blue), and day 150 (pink). The total volatile fraction progressively increases throughout the ripening process, reaching its maximum at day 120, followed by a slight decrease at day 150, thus indicating the presence of dynamic changes in aroma compound formation and transformation during cheese maturation.

**Figure 2:**
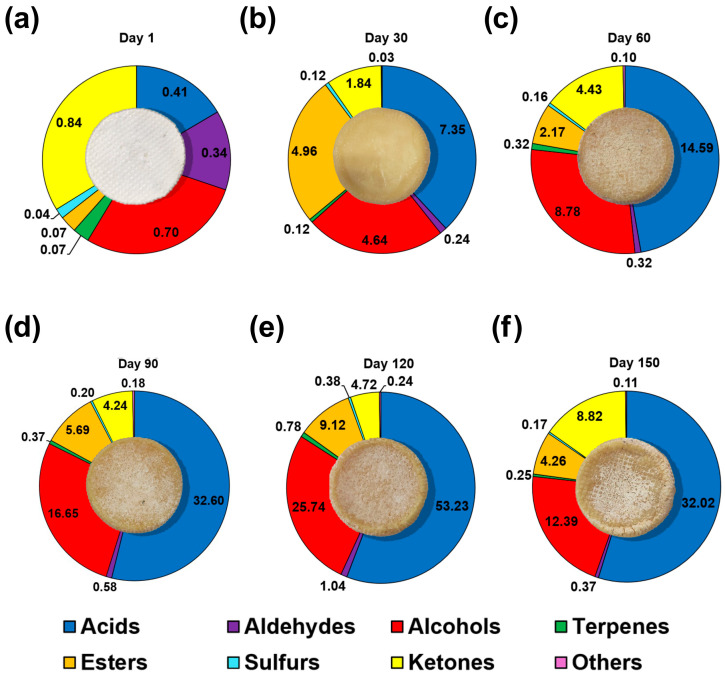
Temporal evolution of surface appearance and VOC classes (mean concentration, mg/kg) during the cheese ripening process at 1 day (**a**), 30 days (**b**), 60 days (**c**), 90 days (**d**), 120 days (**e**), and 150 days (**f**). Pie charts represent the relative contribution of acids (blue), aldehydes (purple), alcohols (red), terpenes (green), esters (orange), sulfur compounds (light blue), ketones (yellow), and other compounds (pink) at each ripening stage. Progressive changes in the cheese color and surface morphology are observable along ripening.

**Figure 3:**
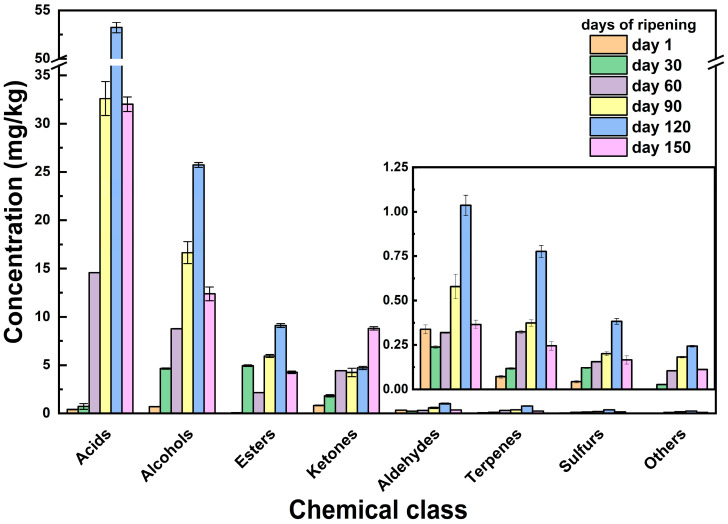
Temporal evolution of the profile of individual VOC classes during the cheese ripening process, including alcohols, ketones, aldehydes, carboxylic acids, terpenes, sulfurs, and others. The bar chart shows the concentration expressed in mg/kg (mean ± standard deviation) of each class at different ripening times (days 1, day 30, day 60, day 90, day 120 and day 150).

Figure 1 reports the total volatile content, while Figure 2 indicates the changes of the main VOC chemical families in the cheese samples across ripening. The total VOC content increased during ripening and peaked around 120 days before slightly declining at 150 days (Figure 2 and Figure 3). This trend reflects intense metabolic activity due to the biochemical progression of lipolysis and proteolysis, which generates precursor molecules for the aroma development along ripening, in line with expected growth of the sensory aroma complexity of mature cheese [10].

### 3.2. Quantitative Evolution of VOCs Throughout Cheese Ripening

Figure 2 and Figure 3 show the changes of the main VOC chemical families during the cheese ripening, revealing the presence of distinct trends among the different classes of compounds as presented below.

#### 3.2.1. Alcohols

Alcohol compounds show a continuous increase from day 1 (0.70 ± 0.02 mg/kg) to 120 days (25.74 ± 0.24 mg/kg), followed by a decrease at 150 days (12.39 ± 0.72 mg/kg) (Table 1; Figure 3). During cheese ripening, alcohols arise from multiple microbial and biochemical pathways, and their temporal evolution reflects the sequential activation of carbohydrate fermentation, amino acid catabolism, and lipid oxidation [26]. The alcohol profile is dominated by the presence of isoamyl alcohol (Alc6) on day 1 and then by ethanol (Alc1) up to 120 days (Table 1). On day 150, 2-butanol (Alc2) is the most representative compound of the alcohol class. This trend suggests the involvement of secondary metabolic pathways, aligning with the fact that secondary alcohols are often found at the highest concentration among all alcohols present at the end of cheese ripening [27]. 2-Butanol is typically formed from diacetyl (2,3-butanedione), especially in raw milk cheeses, which is degraded to acetoin by bacterial enzymes derived from raw milk. Successively, it is reduced to 2,3-butanediol and converted first into 2-butanone, and finally into 2-butanol [28,29,30]. In detail, Ghiaci et al. [29] and Eugster et al. [30] proved that some NSLAB, such as *Pediococcus acidilactici*, can convert meso-2,3-butanediol to 2-butanol, indicating a late-stage metabolic shift in cheese ripening. Also, Wang et al. [31] showed that in Cheddar cheese, the conversion of 2,3-butanediol into 2-butanone and its final reduction into 2-butanol are conducted by a strain of *Lactobacillus brevis*.

In this study, the trend observed for alcohols involves a gradual increase in them up to day 120 followed by a decrease. Similarly to 2-butanol, 2-heptanol (Alc8) constantly increases throughout cheese ripening, while 2-pentanol (Alc4), which is detected only on day 150, deviates from the general trend, emphasizing distinct metabolic dynamics during the late cheese maturation.

#### 3.2.2. Ketones

During cheese ripening, ketones present temporal behavior similar to that observed for alcohols, with the presence of a slight plateau around 60–120 days and a maximum amount of production at 150 days (8.82 ± 0.18 mg/kg) (Table 1). Specifically, acetoin (K7) dominates the VOC profile up to 120 days of ripening, while at the end of the cheese maturation, 2-butanone is the most abundant compound of this class (see Table 1).

In general, during cheese aging, methyl ketones including 2-pentanone (K3), 2-heptanone (K5), and 2-nonanone (K6) display gradual or moderately peaked patterns consistent with typical fatty acid metabolism [32,33]. Within this overall homogeneous behavior, 2-butanone (K2) represents the main exception since it shows an early modest increase and a pronounced late-stage accumulation that made it the dominant ketone in the final stage of maturation.

#### 3.2.3. Aldehydes

During the cheese ripening, aldehydes, present in low amounts at the beginning of the ripening, increase during the mid-ripening phase, reaching the maximum at 120 days (1.04 ± 0.06 mg/kg). On the contrary, aldehydes content diminishes on day 150 (0.36 ± 0.02 mg/kg), in line with the temporal patterns linked to amino acid catabolism and lipid oxidation [34] (Figure 2). In contrast, 2-methylpropanal (Ald1) diverged from this behavior since it appears in the initial stages of the ripening process and disappears from day 60 onward (Table 1). 3-Methylbutanal (Ald3) represents the dominant aldehyde during cheese ripening, with a late-stage maximum (0.29 ± 0.04 mg/kg) followed by a moderate decline at day 150 (0.19 ± 0.01 mg/kg).

#### 3.2.4. Esters

Esters in cheese are formed through esterification reactions between short- and medium-long-chain fatty acids released during milk fat degradation and primary or secondary alcohols produced via lactose fermentation or amino acid metabolism during the ripening process [26,35].

In our experiments, esters show a more complex trend respect to the other VOC families, showing a peak at 30 days (4.96 ± 0.10 mg/kg), followed by a drop, and then a pronounced increase reaching the highest level at 120 days (9.12 ± 0.20 mg/kg) (Table 1 and Figure 2). The most abundant esters across the cheese ripening are ethyl butyrate (E2), ethyl hexanoate (E10), and ethyl octanoate (E15). They show a clear and consistent pattern, with low or absent levels at the start of the ripening, a marked increase during the central stages of the process and a decline toward the end of the ripening (Table 1). Among them, ethyl hexanoate (E10), the main ester along ripening (Table 1), displays the most pronounced dynamics. Its concentration increases sharply during the early stages of ripening (3.85 ± 0.04 mg/kg), followed by a decrease in the middle of the ripening process. This is succeeded by a rise at the late-stage maximum (4.42 ± 0.05 mg/kg), and a final decline at 150 days (2.04 ± 0.03 mg/kg). Ester compounds play a significant role in enhancing the overall cheese aroma, contributing to the sweet, fruity, and floral notes, and reducing the sharpness and bitterness due to the presence of the carboxylic acids [36,37].

#### 3.2.5. Carboxylic Acids

Carboxylic acids represent the most abundant volatile fraction, showing a coherent temporal pattern characterized by a marked increase from 14.59 ± 0.02 mg/kg at 60 days, with a maximum at 120 days (53.22 ± 0.54 mg/kg), followed by a decrease at 150 days, while remaining at relatively high levels (32.02 ± 0.76 mg/kg). These compounds are associated with strong sensory notes described as sweaty, rancid, and cheesy, thereby playing a key role in shaping the intense and complex aroma of mature cheeses [9,38].

Volatile acids originate from the lipolysis and β-oxidation, and their accumulation represents the progressive breakdown of milk fat during aging [3]. Medium-chain acids, such as hexanoic (A7), octanoic (A9), heptanoic (A8), and decanoic (A11), accumulated during the cheese ripening, peaking from the mid-ripening stage. Among them, hexanoic acid is the main acid at each stage of the ripening (Table 1).

Acetic acid (A1) and butanoic acid (A4), the second most abundant acids across ripening, display similar trends, increasing markedly during the central stages of the cheese ripening, and slightly decreasing at the end of the aging process.

#### 3.2.6. Terpenes, Sulfur, and Other Compounds

Although present at low levels, terpenes, sulfur compounds, and other minor volatiles significantly contribute to the aromatic signature of ripened cheeses. Terpenes, such as α-pinene (T1), β-phellandrene/sabinene (T2), p-cymene (T4), and limonene (T3), mainly originate from the animal diet and are transferred to milk, later evolving during cheese ripening to impart resinous, citrus-like, and herbal notes [39,40].

Among sulfur compounds, 4-(methylthio) butanenitrile (S2) and dimethyl sulfone (S3) show trends similar to that of the total VOC content during the cheese ripening, reflecting the presence of established pathways of sulfur-containing amino acid catabolism. These compounds originate from the microbial degradation of methionine and cysteine and are associated with characteristic pungent cabbage- and garlic-like notes [41,42,43,44].

### 3.3. Principal Component Analysis (PCA) and Markers Identification

**Figure 4:**
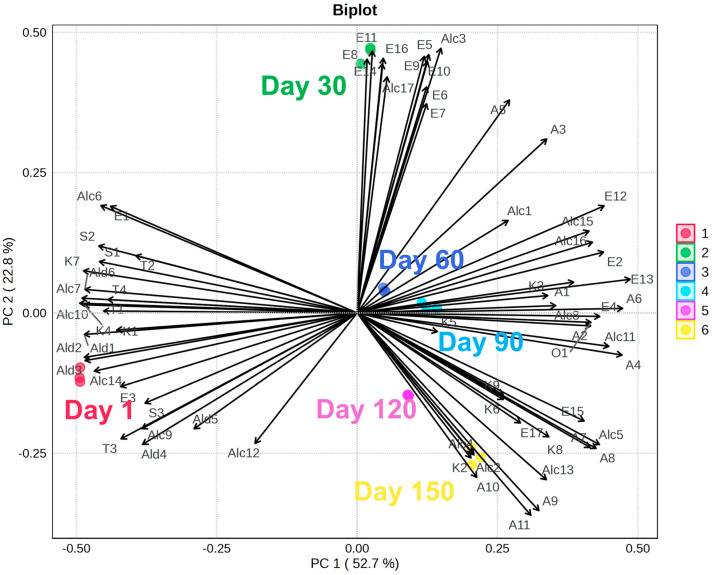
Biplot of the PCA based on the VOC profiles of the goat cheese samples during the cheese ripening process. Cheese samples collected at different ripening times are shown with different colors: day 1 in pink, day 30 in green, day 60 in blue, day 90 in light blue, day 120 in purple, and day 150 in yellow. The first two principal components explain 52.7% (PC1) and 22.8% (PC2) of the total variance, respectively. Arrows represent the loading vectors of individual VOCs, indicating their contribution to sample differentiation and highlighting the progressive temporal shift of the volatile profile along the ripening period.

Figure 4 shows the biplot of the PCA conducted on the VOC profiles obtained by each goat cheese sample along the ripening period The first two principal components explain 75.5% of the total variance. Figure 4 shows clear discrimination among samples according to the dynamic VOC evolution driven by the metabolic activity occurring during maturation.

Cheese samples on day 1 are positioned on the left side of the plot and appear strongly associated with several aldehydes, including 2-methylpropanal (Ald1), 2-methylbutanal (Ald2), 3-methylbutanal (Ald3), nonanal (Ald4), benzaldehyde (Ald5), and phenylacetaldehyde (Ald6). This pattern is consistent with the role of aldehydes as early metabolic products and key intermediates in cheese ripening. These compounds, in fact, are more abundant at the beginning of cheese maturation and subsequently decrease due to their conversion into other volatiles [45] such as isoamyl alcohol (Alc6) [46] and 3-methyl-butyric acid (A5) [47]. Esters, such as ethyl acetate (E1) and ethyl 3-methylbutyrate (E3), as well as ketones, including 2-propanone (K1), diacetyl (K4), and acetoin (K7), are also correlated with the cheese samples at day 1. In particular, the higher relevance at the beginning of ripening of K4 and K7, can be due to the fact that these compounds are early metabolic intermediates and can act as precursors in interconnected pathways for other volatiles, such as 2,3-butanediol across cheese maturation [48,49] and consequently of 2-butanol and 2-butanone [28,29,30,31].

Samples at day 30 move toward the high central region of the biplot and show a clear association with several esters, namely ethyl pentanoate (E5), isobutyl butyrate (E6), ethyl 2-butenoate (E7), pentyl acetate (E8), isobutyl isopentanoate (E9), ethyl hexanoate (E10), ethyl 3-hexenoate (E11), isobutyl hexanoate (E14), and isoamyl hexanoate (E16). This result is directly related to the marked increase in ester compounds occurring at this stage of ripening and suggests the presence of intense microbial activity [50].

At day 60, the cheese samples appear to be mainly associated with ethanol (Alc1), 2-methyl propanoic (A3), and 3-methylbutyric (A5) acid, indicating the presence of enhanced fermentative activity, such as the reduction of aldehydes to alcohols [51], an increase in the amino acid catabolism and the β-oxidation process of fatty acids [52]. At 90 days, the cheese samples are directly correlated to 2-heptanol (Alc8), 2-nonanol (Alc11), 3-(methylthio)-1-propanol (Alc15), and benzyl alcohol (Alc16), and to the esters ethyl butyrate (E2), isoamyl acetate (E4), isoamyl butyrate (E12), and ethyl heptanoate (E13). These findings suggest an increased contribution of secondary metabolic pathways, including amino acid catabolism, lipid oxidation, and esterification reactions [10,52].

At 90 days, 120 days and 150 days, cheese samples are correlated to some ketones, esters and acids, including 2-nonanone (K6), 2-octanone (K8), 8-nonen-2-one (K9), ethyl octanoate (E15), ethyl decanoate (E17), 1-butanol (Alc5), hexanoic (A7), and heptanoic (A8) acid, which have been reported to be a characteristic volatile profile of mid- to fully ripened cheese [53,54].

The most ripened cheese samples, collected at day 120 and day 150, are clearly separated on the right side of PC1. They are mainly associated with octanoic (A9), nonanoic (A10), decanoic (A11) acid, 2-butanone (K2), 2-butanol (Alc2), 2-pentanol (Alc4), and 2,3 butanediol (Alc13), aligning to the accumulation of these specific metabolites in the late ripening stages of fully matured cheese [27,54].

**Figure 5:**
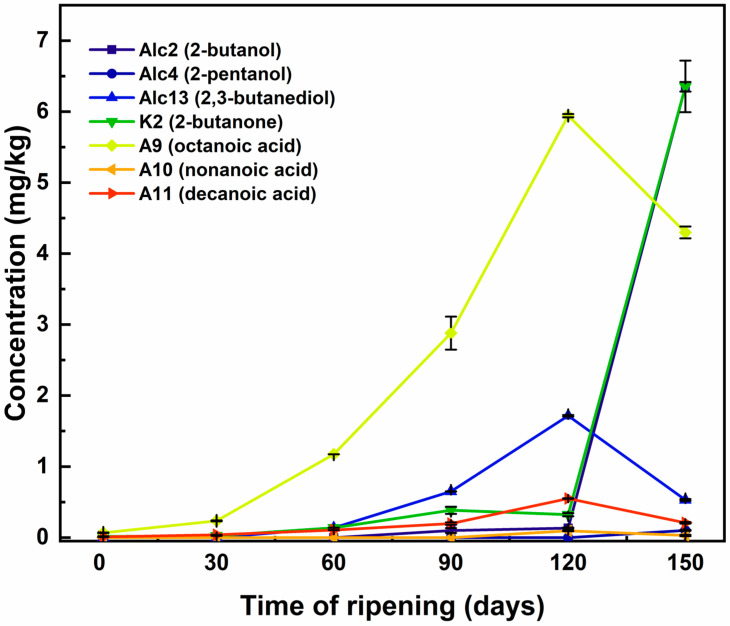
Evolution of discriminant VOCs (Alc2, Alc4, Alc13, K2, A9, A10, and A11) during the cheese ripening process. Concentration trends (mg/kg) identified the discriminant variables associated with the late ripening stage (150 days) according to PCA loadings. Data represents mean values (±standard error).

The combined analysis of VOC dynamics and PCA highlights a specific subset of compounds that are linked to their phase-specific increase during the ripening process. Octanoic (A9), nonanoic (A10), and decanoic acid (A11), 2-butanone (K2), 2-butanol (Alc2), 2-pentanol (Alc4), and 2,3 butanediol (Alc13) show the strongest association with the final ripening stages, as confirmed by their amount trend over time (Figure 5). Specifically, K2 and Alc2 can be considered putative volatile markers of advanced cheese ripening, and their use may be suitable for the design of specific sensors for cheese ripening.

### 3.4. First Warning Sensor

**Figure 6:**
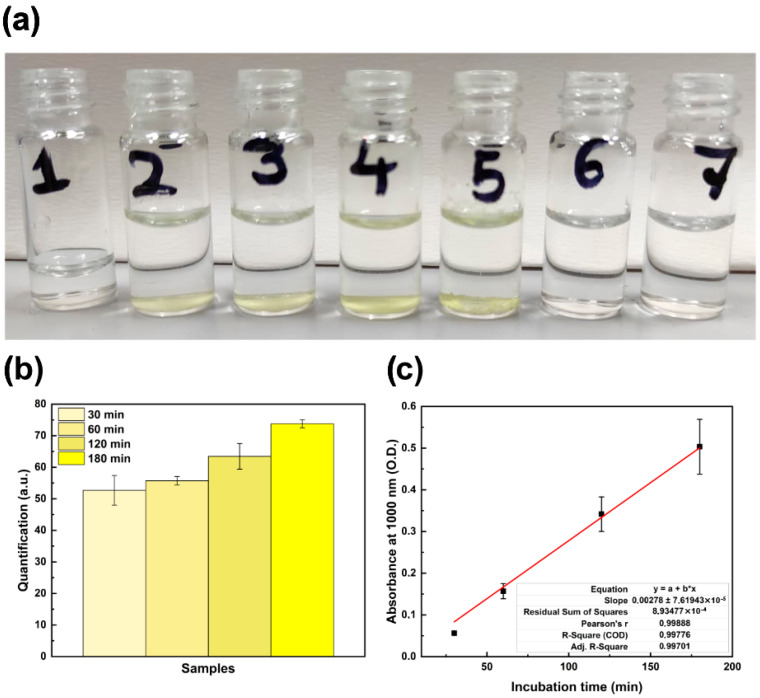
Multi-modal characterization of the iodoform-based chemosensor: (**a**) Iodoform formation in NaOH solutions after exposure to 2-butanone (200 mM) vapors for increasing incubation times of 30 min (2), 1 h (3), 2 h (4), and 3 h (5). Negative controls include NaOH alone (1), NaOH exposed to water vapor (6), and NaOH exposed to ethanol (200 mM) vapor (7). The yellow precipitate indicates a positive iodoform reaction. (**b**) Quantitative digital signal output (Abs (a.u.)) derived from image-based densitometry data. (**c**) Abs at 1000 nm (O.D.) as function of incubation time.

To design a sensing method for the fast detection of cheese ripening, we selected 2-butanone as a marker of the final stage of cheese maturation process, according to the PCA results performed on the HS-SPME/GC-MS data overall obtained. Based on the iodoform test, in the presence of iodine, when a solution of NaOH is exposed to 2-butanone vapors, a yellow-colored compound is formed [19]. As depicted in Figure 6a, the color intensity increases with the exposure time to 2-butanone vapors. After 30 min, a slight turbidity of the solution is observed, indicating the beginning of the reaction. A naked-eye-visible precipitate appears after one hour (Figure 6a), and after three hours it is possible to observe the evident formation of a yellow precipitate, which is not detected in negative controls (NaOH samples exposed to water vapor or ethanol vapor (200 mM)).

To provide analytical measurements of the sensing performance and allow the conversion of the naked-eye colorimetric response into measurable signal outputs, digital densitometry measurements and spectrophotometric analysis were performed.

The quantitative analytical parameters of the chemosensor are summarized in Table 2. In Figure 6b, the quantification (in arbitrary units (a.u.)) of the yellow precipitate in each vial obtained by digital densitometry measurements is reported. A consistent increase (from 52.71 a.u. at 30 min to 73.79 a.u. at 180 min) is observed as a function of the exposure time to 2-butanone vapors.

In Figure 6c, the values of absorbance (Abs) at 1000 nm (O.D.) at different incubation times are reported. The Abs value increases from 0.06 O.D. (at 30 min) to 0.16 O.D. (at 60 min), reaching the value of 0.50 O.D. at 180 min, which is consistent with the exposure time of 2-butanone.

## 4. Conclusions

HS-SPME/GC-MS analysis of the VOC profile of the goat cheese across 150 days of ripening enabled the identification of 68 volatile compounds. The evolution of the VOC pattern revealed distinct biochemical phases, driven by microbial and enzymatic transformations. Carboxylic acids and alcohols dominate the volatile profile of cheese regardless of the ripening process, while esters and ketones, contributing to specific aromatic nuances, followed specific trends. Among the detected compounds, 2-butanone and 2-butanol have been identified as potential volatile markers of the final stage of cheese ripening and are suitable for the design of a sensing method for a rapid and preliminary first screening of the cheese maturation process.

It is important to note that although the proposed method is qualitative and not selective for 2-butanone, which limits its practical applications due to interfering compounds, it provides a simple, low-cost, and easily implementable approach for the early indication of cheese maturation.

## Figures and Tables

**Table 2:** Quantitative analytical parameters of the iodoform-based chemosensor. Data includes the digital colorimetric quantification (a.u.) and spectrophotometric absorbance (O.D.) at 1000 nm. Data is presented as mean values (±standard error (SD)).

Exposure Time (min)	Quantification (a.u.) ± SD	Absorbance at 1000 nm (O.D.) ± SD
30	52.71 ± 4.71	0.06 ± 0.01
60	55.74 ± 1.32	0.16 ± 0.02
120	63.47 ± 4.07	0.34 ± 0.04
180	73.79 ± 1.31	0.50 ± 0.07

## Data Availability

The original contributions presented in this study are included in the article. Further inquiries can be directed to the corresponding author.

## Abbreviations

The following abbreviations are used in this manuscript:

VOCs	Volatile organic compounds
HS-SPME	Headspace solid phase microextraction
GC-MS	Gas chromatography-mass spectrometry
SLAB	Starter lactic acid bacteria
NSLAB	Non-starter lactic acid bacteria
MGV	Mean Gray Value
ROI	Region of Interest
